# Phenotypic Heterogeneity Among Carriers of the Same Pathogenic Variant in Hereditary Hemorrhagic Telangiectasia

**DOI:** 10.3390/genes17091112

**Published:** 2026-09-13

**Authors:** Elena Urízar, Pablo Solis, Nuria Puente, Ana Fontalba, Roberto Zarrabeitia, José A. Riancho

**Affiliations:** 1 Servicio de Medicina Interna, Hospital Universitario Marqués deValdecilla, 39008 Santander, Spain; elena.urizar@scsalud.es (E.U.); pablo.solis@scsalud.es (P.S.); nuria.puente@scsalud.es (N.P.); 2 Instituto de Investigación Marqués de Valdecilla (IDIVAL), 39011 Santander, Spain; 3 Centro de Investigación Biomédica en Red de Enfermedades Raras (CIBERER), 28029 Madrid, Spain; 4 Unidad de Genética, Hospital Universitario Marqués de Valdecilla, 39008 Santander, Spain; anam.fontalba@scsalud.es; 5 Servicio de Medicina Interna, Hospital Sierrallana, 39300 Torrelavega, Spain; roberto.zarrabeitia@scsalud.es; 6 Departamento de Medicina y Psiquiatría, Universidad de Cantabria, 39008 Santander, Spain

**Keywords:** hereditary hemorrhagic telangiectasia, genetics, endoglin, ACVRL1, expressivity, genotype–phenotype correlation, arteriovenous malformations, epistaxis

## Abstract

**Background**: Hereditary hemorrhagic telangiectasia (HHT) is a rare autosomal dominant vascular disorder primarily caused by pathogenic variants in the *ENG* and *ACVRL1* genes. Although genotype–phenotype correlations are well established at the population level, the degree of phenotypic similarity among relatives sharing the same variant is poorly characterized. **Objective**: to explore differences in expressivity and quantify the degree of phenotypic variability among individuals with HHT carrying the same pathogenic variant. **Methods**: We evaluated 60 patients from a reference center with recurrent *ENG* and *ACVRL1* variants, to quantify intra-familial phenotypic heterogeneity using Shannon entropy indices, Jaccard distances, and a latent variable liability-threshold approach. **Results**: There was substantial phenotypic variability among individuals and families, with cutaneous telangiectasia being the most frequent manifestation. Shannon entropy and Jaccard analyses indicated broadly high variability. The shared variant accounted for only 3.3% of total variance. Age was significantly associated with disease manifestations (β = 0.027, *p* = 0.002; OR = 1.028, 95% CI 1.010–1.045), with each additional year increasing the odds of severe symptoms by 2.7%, particularly bleeding severity and skin telangiectasias. **Conclusions**: These findings suggest that the variable expressivity in HHT is predominantly driven by individual-level factors other than the specific pathogenic variant carried by each patient.

## 1. Introduction

Hereditary hemorrhagic telangiectasia (HHT; OMIM #187300 and #600376), also known as Rendu–Osler–Weber syndrome, is a rare autosomal dominant vascular disorder characterized by the development of mucocutaneous telangiectasias and visceral arteriovenous malformations (AVMs). The estimated prevalence ranges from approximately 1 in 5000 to 1 in 8000 individuals worldwide, although considerable geographic variation has been reported due to founder effects and differences in case ascertainment [1]. HHT is associated with substantial morbidity resulting from recurrent epistaxis, gastrointestinal bleeding, iron-deficiency anemia, and potentially life-threatening complications arising from pulmonary, cerebral, or hepatic AVMs.

The disorder is primarily caused by pathogenic variants in genes encoding components of the transforming growth factor-β (TGF-β)/bone morphogenetic protein (BMP) signaling pathway involved in vascular development and endothelial homeostasis. Pathogenic variants in *ENG* and *ACVRL1* account for approximately 85–90% of genetically confirmed cases, while variants in *SMAD4* cause the combined juvenile polyposis/HHT syndrome, and pathogenic variants in *GDF2* and a limited number of additional genes have been identified in rare cases [2,3].

HHT is generally considered to exhibit age-dependent but high penetrance, with most mutation carriers developing characteristic clinical manifestations by adulthood [3]. Nevertheless, disease expressivity is variable. The severity, number, and distribution of vascular lesions, as well as the occurrence of specific organ involvement, differ markedly between affected individuals. This variability has been attributed to a combination of environmental influences, stochastic biological processes, modifier genes, and other yet unidentified factors [2].

Some genotype–phenotype correlations have been described at the population level. Most previous studies have compared patients carrying pathogenic variants in different genes. For example, pulmonary and cerebral AVMs are described more frequently in individuals with *ENG* variants, whereas hepatic vascular malformations predominate in those with *ACVRL1* variants [4,5,6]. In contrast, relatively little attention has been paid to the degree of phenotypic similarity among relatives carrying the same pathogenic variant.

Determining whether clinical manifestations cluster within families or vary predominantly between individuals is pertinent not only from a scientific perspective. It has important implications for genetic counseling, as it may improve the ability to counsel relatives carrying the same pathogenic variant about the expected spectrum and predictability of disease manifestations in their offspring [7,8].

The present study aimed to quantify the degree of phenotypic variability among individuals with HHT carrying the same pathogenic variant. Specifically, we used multilevel generalized linear mixed-effects models to partition the variability in the occurrence of major HHT manifestations into family- and individual-level components, thereby assessing the extent to which clinical expression is shared among carriers of the same variant.

## 2. Materials and Methods

We searched the database of patients followed up at the HHT clinic of Hospital Universitario Marqués de Valdecilla, the reference hospital in Cantabria, a region in Northern Spain with a population of around 560,000. We identified patients carrying recurrent genetic variants of *ENG* and *ACVRL1* present in four or more individuals. The study group included 60 patients, 3 *ENG* recurrent variants and 4 *ACVRL1* variants (Table 1). For the purposes of this study, all individuals carrying the same mutation were considered as a “family”. The study was approved by the IRB (CEIMc acta 18/2025).

We reviewed the electronic health records and extracted data about 5 manifestations covering the clinical spectrum of HHT: (1) the global hemorrhagic severity score recently proposed by Al-Samkari, which integrates the solitary hematologic measurements (hemoglobin, intravenous iron infused, and red cells transfused) and classifies the severity of bleeding as mild, moderate, severe or very severe [7,8]; (2) presence of cutaneous telangiectasia; (3) presence of lung AVM; (4)presence of cerebral AVMs; and (5) presence of liver vascular abnormalities. For the purposes of the study, the severity score was categorized as moderate or more than moderate. Visceral AVMs were diagnosed by lung CT scan, brain angio-CT or angio-MRI, and Doppler ultrasound and/or CT of the liver. In all cases, experienced radiologists analyzed the studies, and the findings were categorized as presence or absence of AVMs.

The variability in clinical expression within each genetic variant was first assessed by visual inspection of the heat maps. Then several quantitative procedures were used. Thus, for each of the 5 clinical manifestations, we computed the Shannon entropy index, and the Jaccard’s distances. Shannon’s entropy index (H) is a measure of diversity that quantifies the uncertainty in predicting the category of a randomly selected individual from a population, incorporating both the number of categories and their relative frequencies [9].

For the case of a binary variable, it can be computed as:

H=−[pxlog2(p)+(1−p)xlog2(1−p)]

where *p* is the frequency of the manifestation.

The scale goes from 0 to 1 and is maximum when subjects are distributed equally (in the case of binary variables, when 50% of subjects belong to each category), and minimum when all individuals belong to the same category.

Jaccard distances (dJ) [10] were calculated to quantify phenotypic dissimilarity between individuals within each genetic variant group. For each pairwise comparison, the Jaccard distance was defined as

$$dJ=1-\left[\frac{m11}{m11+m10+m01}\right]$$

where m11 represents shared presence of a phenotype and m10 and m01 represent discordant phenotype presence between individuals. The shared absences were excluded.

However, the distances were also computed including the shared absences, with the formula

$$dJ=1-\left[\frac{m11+m00}{m11+m00+m10+m01}\right]$$

For relatively common phenotypic features, both approaches can provide complementary information and may be considered in the assessment of intra-familial phenotypic similarity, with a scale ranging from 0 to 1. Missing values were treated as unavailable data and excluded pairwise rather than being imputed as absent. Mean intra-familial Jaccard distances were then calculated to estimate phenotypic variability within each group of carriers of a given genetic variant, with lower values indicating phenotypic homogeneity and higher values indicating increased phenotypic heterogeneity.

We used the latent variable (or Liability-Threshold) approach used to partition variance and estimate the Intraclass correlation coefficient (ICC) for binary data. In this approach, the binary manifestations are assumed to be driven by an unobserved, continuous latent propensity (the logistic scale). Thus, a composite familial phenotypic variability index was estimated by pooling the five binary symptoms across individuals carrying the same genetic variant. For each “family” (this is, group of subjects with the same variant), a symptom presence proportion was calculated as the number of observed symptom-positive features divided by the total number of valid symptom observations, with missing values excluded. Variability between families was quantified as the variance of these family-level proportions and transformed to the logistic scale using the global symptom prevalence. An ICC was subsequently estimated as the ratio of between-family variance to total latent-scale variance, assuming a logistic residual variance of Π^2^/3 (≈3.29). Phenotypic variability was additionally assessed at the individual symptom level. For each binary symptom, family-specific prevalence was calculated. Between-family variance was estimated from the distribution of symptom-specific family prevalences and transformed to the logistic scale. An ICC-like measure was then calculated as before.

To jointly analyze the symptoms while accounting for the hierarchical structure of the data, we fitted generalized linear mixed-effects models (GLMMs) with a binomial distribution and logit link [11,12]. Unlike the prior approach that aggregates individual binary data up to a single family-level summary metric first (the “composite index” or proportion), GLMMs handle the nested hierarchy (individuals clustered inside families. GLMMs combine fixed effects, which estimate the association between predictors and the outcome for the entire population, with random effects, which account for variability between clusters (e.g., families or individuals). By incorporating random effects, GLMMs appropriately model the dependence among observations within the same cluster and provide unbiased estimates of both the effects of interest and the variance attributable to different hierarchical levels. Symptom type (five categories) was included as a fixed effect to account for differences in the baseline prevalence of the individual symptoms, while random intercepts were specified at both the family and individual levels to model between-family and between-individual variability. Age and sex were subsequently included as fixed effects to evaluate their association with symptom occurrence after adjustment for symptom type. The primary objective of the analysis was to partition the variability in symptom occurrence into family-level and individual-level components while simultaneously estimating the effects of symptom type, and the covariates’ age and sex. ICCs were calculated on the latent logistic scale using the estimated random-effect variances and the standard logistic residual variance (π^2^/3). The family-level ICC quantified the correlation in symptom occurrence between different individuals sharing the same genetic variant, whereas the within-individual ICC quantified the correlation between different symptoms measured in the same individual.

Generative AI software (ChatGPT 5.6 Luna and Google Gemini, August 2026 version, Google DeepMind (London, UK)) was used to help to write the scripts for the statistical analyses and to improve the syntax and clarity of the manuscript. These analyses were carried out with dedicated scripts run in SPSS software for Windows v.27.

## 3. Results

The genetic variants studied and the main clinical characteristics are shown in Table 1.

**Figure 1:**
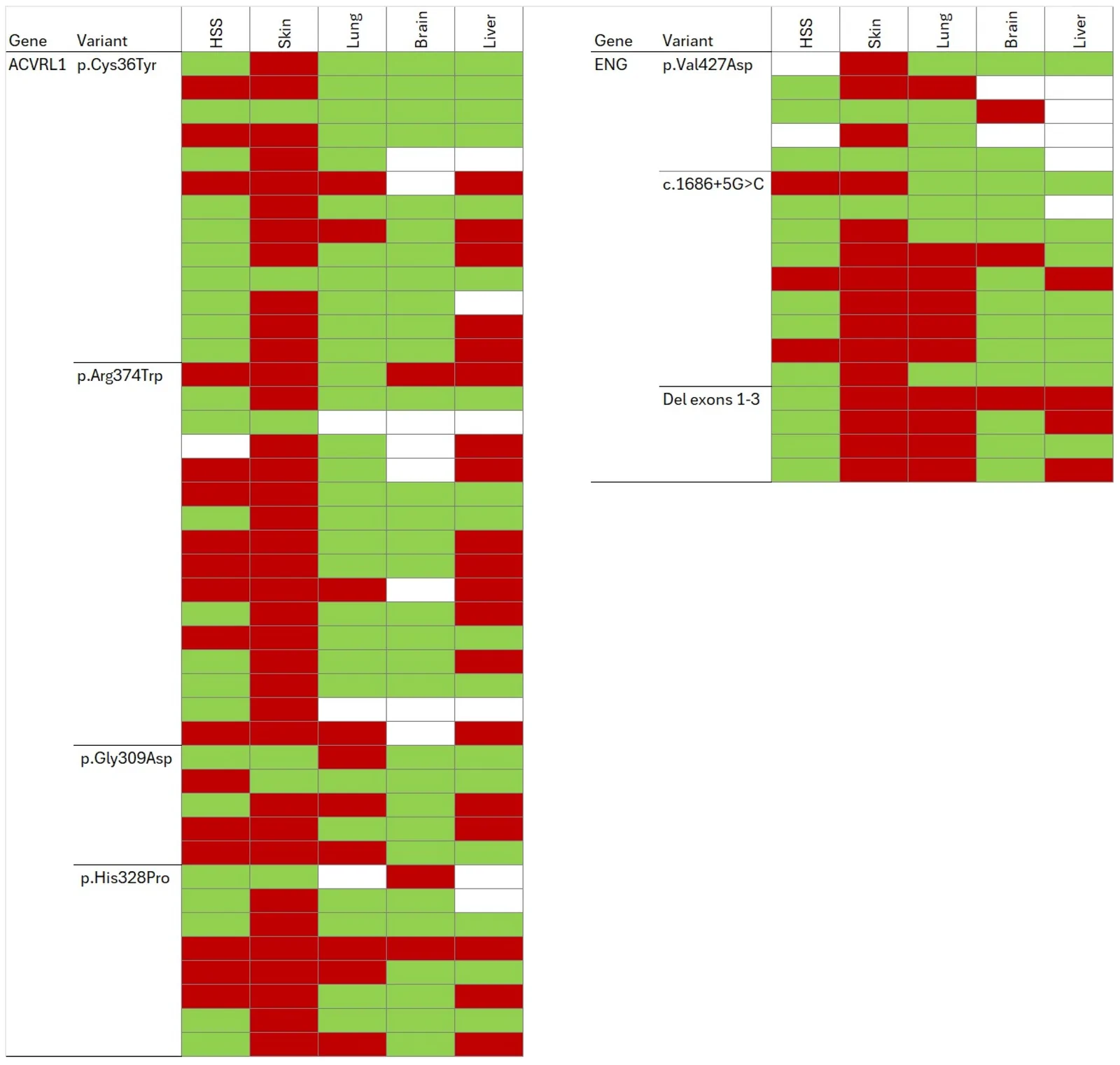
Heat map showing the presence (red) or absence (green) of manifestations. Each row represents an individual. White cells indicate absence of reliable information. HSS, hemorrhagic severity score.

**Figure 2:**
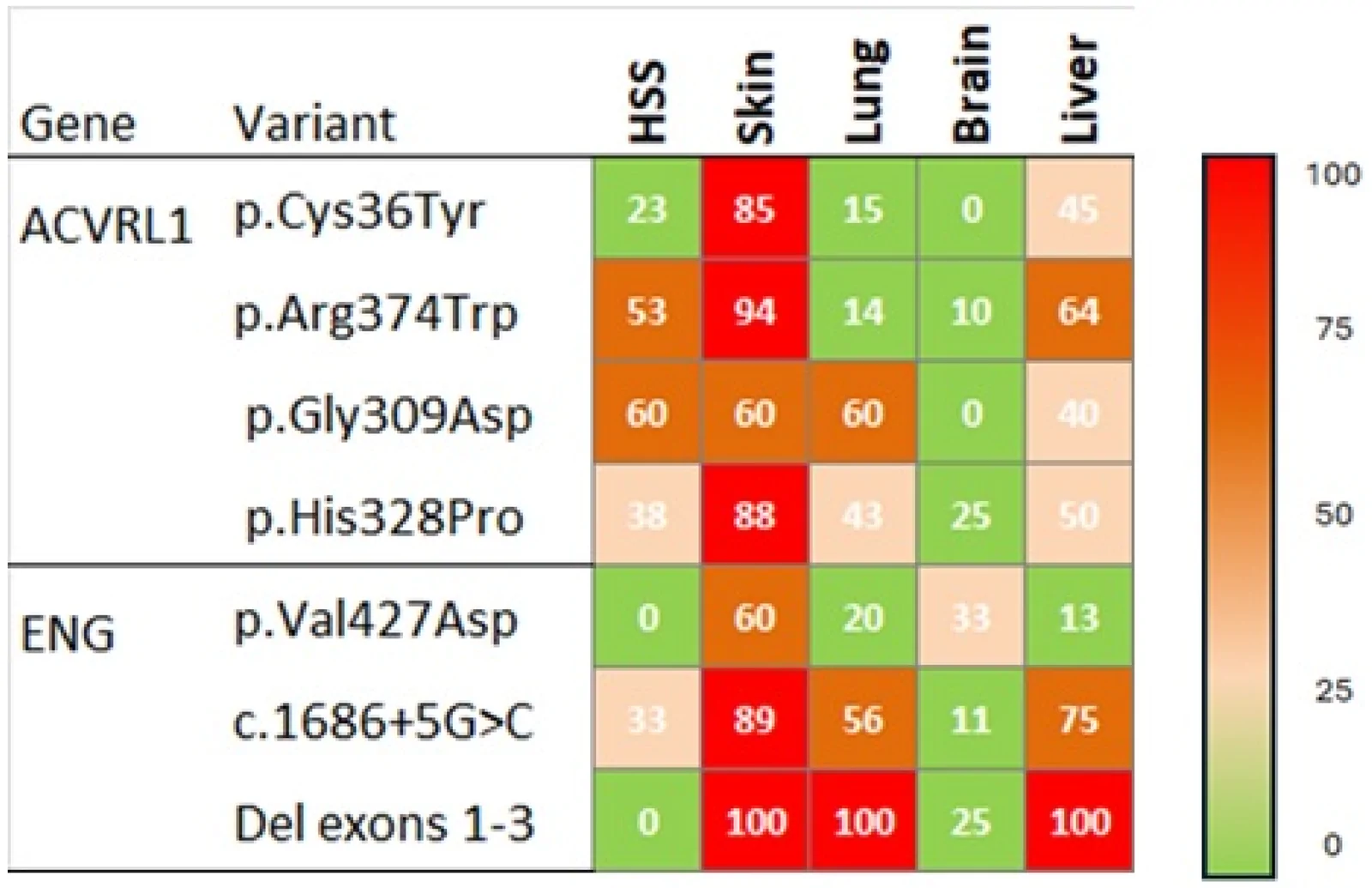
Aggregated frequencies of the manifestations in groups of subjects sharing the same genetic variant. HSS, haemorrhagic severity score.

The individual manifestations are shown at the subject level in Figure 1 and as aggregated frequencies in Figure 2. The inspection of the heat maps reveals that cutaneous telangiectasia was most frequent. Likewise, clinical manifestations varied both among subjects carrying different genetic variants and among those carrying the same variant.

As a numerical quantification of variability, we first computed the Shannon entropy index. As shown in Table 2, the Shannon index was generally high, ranging from 0.41 through 0.82 across the manifestations. It was higher within ACVRL1 variants than within ENG variants regarding 3 manifestations (hemorrhagic severe score, presence of pulmonary AVMs, and presence of liver vascular abnormalities). It was similar in both for one manifestation (skin telangiectasia), and higher among ENG variants regarding the presence of central nervous system AVMs.

To analyze the overall intrafamilial variability, we used several alternative approaches, including Jaccard distances, composite phenotypic variability and generalized mixed models.

First, we computed Jaccard distances. The intrafamilial Jaccard distances, either classical or modified, were slightly higher among carriers of *ACVRL1* variants than among those with *ENG* variants (Table 3). However, differences in family size must be taken into account.

Second, a composite familial phenotypic variability index was estimated by pooling the five binary symptoms across individuals within each family. Among 273 valid observations, 120 (44%) indicated the presence of a manifestation. The ICC, representing the between-family variation, was only 1.3% (0.6% among carriers of *ACVRL1* variants and 2.9% among carriers of *ENG* variants). In other words, 98.7% of the variation could not be attributed to the type of genetic variant. In line with those results, the analysis of individual symptoms showed that the vast majority of the variability (93–99% for *ACVRL1* variant carriers, and 81–97% for carriers of *ENG* variants) was not explained by the type of variant.

Third, a generalized linear mixed-effects logistic model was then used to jointly analyze the five binary symptoms, accounting for clustering at the family and individual levels.

The analysis of the random effects revealed low familial aggregation for symptom severity; the shared family structure accounted for only 3.3% of the total latent variance ICC = 0.033). Conversely, individual patient characteristics nesting within families explained 9.8% of the variance, indicating a modest-to-low intra-subject correlation across the five evaluated features (ICC = 0.131). The vast majority of the underlying variability (86.9%) resided in the intrinsic logistic residual variance. Regarding fixed effects, a significant association was observed for chronological age (β = 0.027, *p* = 0.002; OR = 1.028, 95% CI 1.010–1.045), demonstrating that each additional year of age is associated with a 2.7% increase in the odds of experiencing severe symptoms. When we focused the analysis on individual symptoms, age remained significantly associated with the severity of bleeding and the presence of skin telangiectasias. On the other hand, no statistically significant disparities were detected for biological sex (β = 0.607; *p* = 0.093).

## 4. Discussion

In this study, we quantified the degree of phenotypic variability among individuals with HHT carrying the same pathogenic variant using complementary analytical approaches, including entropy measures, Jaccard distances, ICCs, and multilevel generalized linear mixed models. Despite sharing the same causal pathogenic variant, affected individuals exhibited substantial heterogeneity in the occurrence of the major clinical manifestations of HHT. Across all analytical methods, the contribution of family-related factors to phenotypic variability was consistently small, whereas most variability occurred at the individual level. Only a small proportion of the total phenotypic variability (0.4–3.3%, depending on the analytical approach) was attributable to family-level clustering among individuals sharing the same pathogenic variant.

These findings reinforce the concept that HHT, although considered a highly penetrant monogenic disorder, is characterized by markedly variable expressivity [3]. Previous studies have consistently demonstrated genotype–phenotype correlations, with pulmonary and cerebral AVMs occurring more frequently in individuals carrying *ENG* variants, and hepatic vascular malformations predominating among carriers of *ACVRL1* variants [1,13]. However, these associations describe average differences between genes rather than the variability observed among individuals carrying the same pathogenic variant. Our results extend previous observations [14] by quantitatively demonstrating that substantial phenotypic heterogeneity persists within carriers of identical variants. The limited sample size of our study did not allow us to explore the role of the variant type (i.e., protein-truncating or non-truncating), which has recently been suggested as another predictor of disease severity [15].

The biological basis of this variability remains incompletely understood. Several mechanisms have been proposed, including the influence of other genetic loci, epigenetic regulation, stochastic developmental events, environmental exposures, and local vascular factors affecting angiogenesis and endothelial homeostasis [16,17]. The remarkably low family-level ICC observed in the present study suggests that the pathogenic variant alone accounts for only a small fraction of the overall variability in clinical expression. Instead, our findings support a model in which individual-specific determinants play a predominant role in shaping the HHT phenotype. This interpretation is also consistent with experimental evidence indicating that vascular malformations frequently require additional local events beyond the inherited germline mutation, in accordance with the proposed “second-hit” mechanism [18,19].

Although age was independently associated with symptom occurrence, reflecting the well-recognized age-dependent penetrance of HHT [13,20], adjustment for age and sex only modestly reduced the unexplained individual-level heterogeneity. This observation suggests that demographic factors explain only a small proportion of the clinical variability and that differences among affected individuals remain largely attributable to other biological mechanisms. Sex was not significantly associated with the composite phenotype, although sex-specific effects have been reported for individual manifestations such as hepatic vascular malformations in previous studies.

Our study has several strengths. To our knowledge, this is one of the first studies to formally quantify intrafamilial phenotypic variability in HHT using complementary statistical methodologies. Rather than relying exclusively on descriptive comparisons, we combined entropy-based measures, similarity indices, variance partitioning, and multilevel generalized linear mixed models to evaluate phenotypic concordance from different perspectives. The consistency of the findings across these independent approaches increases confidence in the robustness of the conclusions.

Several limitations should also be acknowledged. First, the study was conducted in a single cohort, and replication in independent populations will be necessary to confirm the generalizability of the findings. Second, some pathogenic variants were represented by only a small number of individuals, limiting the precision of family-specific estimates. Third, the analysis was based on a limited number of binary clinical manifestations and therefore did not capture all potential differences in disease severity, lesion burden, age at onset, or longitudinal progression. Fourth, given the retrospective nature of the study, the incomplete data may have influenced the precision of some estimates, particularly in the case of the p.Val427Asp *ENG* variant. A further limitation is that the clustering variable was defined by groups of individuals sharing the same variant rather than by the degree of biological relatedness. Because closely related individuals share a larger proportion of their genetic background and, in some cases, environmental exposures, they may exhibit greater phenotypic similarity than more distant relatives. Our approach therefore provides an estimate of overall phenotypic clustering among carriers of the same pathogenic variant but may underestimate familial concordance within closely related pedigrees. Lastly, since we just analyzed 4 *ACVRL1* variants and 3 *ENG* variants, we do not know if the results are similar across the whole mutational spectrum of HHT.

Nevertheless, from a clinical perspective, these findings have important implications for genetic counseling and patient management. Relatives carrying the same pathogenic variant cannot be assumed to develop similar patterns of organ involvement or disease severity based on the phenotype of another affected family member. Although knowledge of the causal gene remains valuable for estimating average risks of specific manifestations, the feasibility of predicting the individual clinical phenotype appears to be limited. Consequently, comprehensive clinical screening according to current HHT guidelines [21,22] remains essential for all mutation carriers, irrespective of the manifestations observed in other affected relatives. More broadly, the results emphasize that monogenic inheritance does not necessarily imply phenotypic predictability.

## 5. Conclusions

Overall, our findings demonstrate that the variable expressivity in HHT is predominantly driven by individual-level factors other than the specific pathogenic variant carried by each patient. They highlight the need for future studies to identify additional genetic, molecular, and environmental modifiers of disease expression, as well as biomarkers [23], ultimately improving individualized risk prediction and personalized management of patients with HHT.

## Figures and Tables

**Table 1:** Variants, number of individuals, sex and age.

Gene	Variant	Patients (n)	Sex (Male/Female)	Age, yr (Mean, Range)
***ACVRL1***	p.Cys36Tyr	13	4/9	45 (19–83)
	p.Arg374Trp	16	7/9	48 (8–82)
	p.Gly309Asp	5	1/4	33 (16–55)
	p.His328Pro	8	4/4	46 (22–69)
***ENG***	p.Val427Asp	5	2/3	44 (9–82)
	c.1686+5G>C	9	6/3	50 (11–87)
	Del exons 1–3	4	2/2	40 (26–55)

**Table 2:** Shannon entropy index. Frequency refers to the frequency (%) of the manifestation among carriers of the variant. HSS, hemorrhagic severity score. AVMs, arteriovenous malformations. VMs, vascular malformations.

		HSS		Telangiectasia		Lung		Brain		Liver	
Gene	Variant	Frequency	Entropy	Frequency	Entropy	Frequency	Entropy	Frequency	Entropy	Frequency	Entropy
*ACVRL1*	p.Cys36Tyr	23	0.78	85	0.62	15	0.62	0	0.00	45	0.99
	p.Arg374Trp	53	1.00	94	0.34	14	0.59	10	0.47	64	0.94
	p.Gly309Asp	60	0.97	60	0.97	60	0.97	0	0.00	40	0.97
	p.His328Pro	38	0.95	88	0.54	43	0.99	25	0.81	50	1.00
	Mean ± SD	43 ± 24	0.93 ± 0.46	81 ± 16	0.62 ± 0.34	33 ± 31	0.79 ± 0.35	9 ± 13	0.32 ± 0.38	50 ± 27	0.98 ± 0.37
											
*ENG*	p.Val427Asp	0	0.00	60	0.97	20	0.72	33	0.92		
	c.1686+5G>C	33	0.92	89	0.50	56	0.99	11	0.50	13	0.54
	Del exons 1-3	0	0.00	100	0.00	100	0.00	25	0.81	75	0.81
	Mean ± SD	20 ± 19	0.32 ± 0.53	73 ± 21	0.65 ± 0.49	60 ± 40	0.56 ± 0.51	19 ± 11	0.58 ± 0.22	38 ± 40	0.59 ± 0.41
											
GLOBAL	Mean ± SD	0.30 ± 0.24	0.66 ± 0.46	0.82 ± 0.16	0.56 ± 0.34	0.44 ± 0.31	0.70 ± 0.35	0.15 ± 0.13	0.50 ± 0.38	0.41 ± 0.27	0.75 ± 0.37

**Table 3:** Average Jaccard distances of the 5 manifestations within genetic variants.

Gene	Variant	Jaccard Distance	Modified Jaccard Distance *
*ACVRL1*	p.Cys36Tyr	0.564	0.301
	p.Arg374Trp	0.467	0.359
	p.Gly309Asp	0.717	0.48
	p.His328Pro	0.621	0.48
	Mean ± SD	0.592 ± 0.104	0.405 ± 0.090
*ENG*	p.Val427Asp	0.8	0.458
	c.1686+5G>C	0.605	0.362
	Del exons 1–3	0.278	0.2
	Mean ± SD	0.561 ± 0.264	0.340 ± 0.130

(*) Considering shared absences.

## Data Availability

Raw data and scripts are available from the authors upon reasonable request.
